# Supplementary material for: HIVprotI: an integrated web based platform for prediction and design of HIV proteins inhibitors
Source: J Cheminform. 2018 Mar 9;10:12. doi: 10.1186/s13321-018-0266-y (PMC5845081; doi:10.1186/s13321-018-0266-y)
Supplement: Supplementary file 2 — Additional file 2. Source code of HIVProtI web server. [file 13321_2018_266_MOESM2_ESM.zip › HIVprotI_Source-code/hivprotI/weka-3-6-6/doc/weka/core/converters/AbstractLoader.html]

AbstractLoader


---


|  |  |  |
| --- | --- | --- |
| **Overview** \| **Package** \| **Class** \| **Tree** \| **Deprecated** \| **Index** \| **Help** \| **Weka's home** | |  |
| **PREV CLASS**   **NEXT CLASS** | **FRAMES**    **NO FRAMES**     **All Classes** |  |
| SUMMARY: NESTED \| FIELD \| CONSTR \| METHOD | DETAIL: FIELD \| CONSTR \| METHOD |  |


---


## weka.core.converters Class AbstractLoader

```
java.lang.Object
  weka.core.converters.AbstractLoader
```

**All Implemented Interfaces:**: java.io.Serializable, Loader, RevisionHandler

**Direct Known Subclasses:**: AbstractFileLoader, DatabaseLoader, TextDirectoryLoader

---

``` public abstract class AbstractLoader extends java.lang.Object implements Loader ```

Abstract class gives default implementation of setSource
methods. All other methods must be overridden.

**Version:**
:   $Revision: 7454 $

**Author:**
:   Richard Kirkby (rkirkby@cs.waikato.ac.nz)

**See Also:**: Serialized Form

---

**Field Summary**

**Fields inherited from interface weka.core.converters.Loader**

`BATCH, INCREMENTAL, NONE`


**Constructor Summary**

`AbstractLoader()`


| **Method Summary** | |
| --- | --- |
| `abstract  Instances` | `getDataSet()`             Return the full data set. |
| `abstract  Instance` | `getNextInstance(Instances structure)`             Read the data set incrementally---get the next instance in the data set or returns null if there are no more instances to get. |
| `abstract  Instances` | `getStructure()`             Determines and returns (if possible) the structure (internally the header) of the data set as an empty set of instances. |
| `void` | `reset()`             Default implementation sets retrieval mode to NONE |
| `void` | `setRetrieval(int mode)`             Sets the retrieval mode. |
| `void` | `setSource(java.io.File file)`             Default implementation throws an IOException. |
| `void` | `setSource(java.io.InputStream input)`             Default implementation throws an IOException. |

**Methods inherited from class java.lang.Object**

`equals, getClass, hashCode, notify, notifyAll, toString, wait, wait, wait`

**Methods inherited from interface weka.core.RevisionHandler**

`getRevision`

**Constructor Detail**

### AbstractLoader

```
public AbstractLoader()
```


**Method Detail**

### setRetrieval

```
public void setRetrieval(int mode)
```

:   Sets the retrieval mode.

    :   **Specified by:**: `setRetrieval` in interface `Loader`
    :   **Parameters:**: `mode` - the retrieval mode

---


### setSource

```
public void setSource(java.io.File file)
               throws java.io.IOException
```

:   Default implementation throws an IOException.

    :   **Specified by:**: `setSource` in interface `Loader`
    :   **Parameters:**: `file` - the File **Throws:**: `java.io.IOException` - always

---


### reset

```
public void reset()
           throws java.lang.Exception
```

:   Default implementation sets retrieval mode to NONE

    :   **Specified by:**: `reset` in interface `Loader`
    :   **Throws:**: `never.`: `java.lang.Exception` - if Loader can't be reset for some reason.

---


### setSource

```
public void setSource(java.io.InputStream input)
               throws java.io.IOException
```

:   Default implementation throws an IOException.

    :   **Specified by:**: `setSource` in interface `Loader`
    :   **Parameters:**: `input` - the input stream **Throws:**: `java.io.IOException` - always

---


### getStructure

```
public abstract Instances getStructure()
                                throws java.io.IOException
```

:   **Description copied from interface: `Loader`**
:   Determines and returns (if possible) the structure (internally the
    header) of the data set as an empty set of instances.

    :   **Specified by:**: `getStructure` in interface `Loader`
    :   **Returns:**: the structure of the data set as an empty set of Instances **Throws:**: `java.io.IOException` - if there is no source or parsing fails ``` public_normal_behavior requires: model_sourceSupplied == true && model_structureDetermined == false && (* successful parse *); modifiable: model_structureDetermined; ensures: \result != null && \result.numInstances() == 0 && model_structureDetermined == true; also public_exceptional_behavior requires: model_sourceSupplied == false || (* unsuccessful parse *); signals: (IOException); ```

---


### getDataSet

```
public abstract Instances getDataSet()
                              throws java.io.IOException
```

:   **Description copied from interface: `Loader`**
:   Return the full data set. If the structure hasn't yet been determined
    by a call to getStructure then the method should do so before processing
    the rest of the data set.

    :   **Specified by:**: `getDataSet` in interface `Loader`
    :   **Returns:**: the full data set as an Instances object **Throws:**: `java.io.IOException` - if there is an error during parsing or if getNextInstance has been called on this source (either incremental or batch loading can be used, not both). ``` public_normal_behavior requires: model_sourceSupplied == true && (* successful parse *); modifiable: model_structureDetermined; ensures: \result != null && \result.numInstances() >= 0 && model_structureDetermined == true; also public_exceptional_behavior requires: model_sourceSupplied == false || (* unsuccessful parse *); signals: (IOException); ```

---


### getNextInstance

```
public abstract Instance getNextInstance(Instances structure)
                                  throws java.io.IOException
```

:   **Description copied from interface: `Loader`**
:   Read the data set incrementally---get the next instance in the data
    set or returns null if there are no
    more instances to get. If the structure hasn't yet been
    determined by a call to getStructure then method should do so before
    returning the next instance in the data set.
    If it is not possible to read the data set incrementally (ie. in cases
    where the data set structure cannot be fully established before all
    instances have been seen) then an exception should be thrown.

    :   **Specified by:**: `getNextInstance` in interface `Loader`
    :   **Parameters:**: `structure` - the dataset header information, will get updated in case of string or relational attributes **Returns:**: the next instance in the data set as an Instance object or null if there are no more instances to be read **Throws:**: `java.io.IOException` - if there is an error during parsing or if getDataSet has been called on this source (either incremental or batch loading can be used, not both).


---


|  |  |  |
| --- | --- | --- |
| **Overview** \| **Package** \| **Class** \| **Tree** \| **Deprecated** \| **Index** \| **Help** \| **Weka's home** | |  |
| **PREV CLASS**   **NEXT CLASS** | **FRAMES**    **NO FRAMES**     **All Classes** |  |
| SUMMARY: NESTED \| FIELD \| CONSTR \| METHOD | DETAIL: FIELD \| CONSTR \| METHOD |  |


---
